# Temporal genetic variation of the red fox, *Vulpes vulpes*, across western Europe and the British Isles

**DOI:** 10.1016/j.quascirev.2012.10.010

**Published:** 2012-12-04

**Authors:** Ceiridwen J. Edwards, Carl D. Soulsbury, Mark J. Statham, Simon Y.W. Ho, Dave Wall, Gaudenz Dolf, Graziella Iossa, Phillip J. Baker, Stephen Harris, Benjamin N. Sacks, Daniel G. Bradley

**Affiliations:** a Smurfit Institute of Genetics, Trinity College, Dublin 2, Ireland; b Research Laboratory for Archaeology, University of Oxford, Dyson Perrins Building, South Parks Road, Oxford OX1 3QY, UK; c School of Biological Sciences, University of Bristol, Woodland Road, Bristol BS8 1UG, UK; d School of Life Sciences, University of Lincoln, Brayford Pool, Lincoln LN6 7TS, UK; e Canid Diversity and Conservation Laboratory, Center for Veterinary Genetics, University of California, One Shields Avenue/Old Davis Road, Davis, CA 95616-8744, USA; f School of Biological Sciences, University of Sydney, NSW 2006, Sydney, Australia; g UCD School of Biology and Environmental Science, University College Dublin, Ireland; h Institute of Genetics, Vetsuisse Faculty, University of Berne, 3001 Berne, Switzerland; i Department of Population Health and Reproduction, School of Veterinary Medicine, University of California, One Shields Avenue/Old Davis Road, Davis, CA 95616-8744, USA

**Keywords:** Red fox, Mitochondrial DNA, Cytochrome *b* gene, Control region, Phylogeography, Europe, British Isles

## Abstract

Quaternary climatic fluctuations have had profound effects on the phylogeographic structure of many species. Classically, species were thought to have become isolated in peninsular refugia, but there is limited evidence that large, non-polar species survived outside traditional refugial areas. We examined the phylogeographic structure of the red fox (*Vulpes vulpes*), a species that shows high ecological adaptability in the western Palaearctic region. We compared mitochondrial DNA sequences (cytochrome *b* and control region) from 399 modern and 31 ancient individuals from across Europe. Our objective was to test whether red foxes colonised the British Isles from mainland Europe in the late Pleistocene, or whether there is evidence that they persisted in the region through the Last Glacial Maximum.

We found red foxes to show a high degree of phylogeographic structuring across Europe and, consistent with palaeontological and ancient DNA evidence, confirmed via phylogenetic indicators that red foxes were persistent in areas outside peninsular refugia during the last ice age. Bayesian analyses and tests of neutrality indicated population expansion. We conclude that there is evidence that red foxes from the British Isles derived from central European populations that became isolated after the closure of the landbridge with Europe.

## Highlights

► Red foxes show a high degree of phylogeographic structuring across Europe. ► Foxes were persistent in areas outside peninsular refugia during the last ice age. ► British foxes originated in central Europe, becoming isolated after 8200 BP.

## 1 Introduction

Studies of ancient DNA have suggested that there was relatively little phylogeographical structuring of species in Europe prior to the last Quaternary glaciation (Hofreiter et al., 2004). Therefore, it appears that cycles of retreat into refugial areas during glacial periods, followed by incomplete dispersal from one refugium into another during interglacial periods, are responsible for the deep genetic divergences now seen between peninsular regions in southern Europe. The Balkans, Iberia and Italy have been identified as refugia where flora and fauna survived during glacial phases (Taberlet et al., 1998; Hewitt, 2000, 2004; Randi, 2007). However, several recent studies have indicated that this ‘southern refugial model’ (Bhagwat and Willis, 2008) is probably too simplistic and not satisfactory for all taxa. Alternative concepts have been developed involving the existence of northern refugia (Stewart and Lister, 2001; Kotlik et al., 2006; Bhagwat and Willis, 2008; Tougard et al., 2008), microrefugia (small favourable areas outside the main refugium; Rull, 2009), and nunatak versus lowland refugia (specific to mountain species; Holderegger and Thiel-Egenter, 2009). As habitat modelling suggests that boreal and tundra habitat extended across central Europe as far north as England (Hewitt, 2000; Fløjgaard et al., 2009), it is also possible that some ecologically adaptable species (those species that are capable of living in various habitats) were not restricted to refugial areas at all during the Last Glacial Maximum (LGM).

Evidence in a range of species seems to support this absence of refugial populations (see Bhagwat and Willis, 2008). One example is the brown bear (*Ursus arctos*), which was typically thought to be restricted to peninsular refugia (Taberlet et al., 1998). However, Valdiosera et al. (2007) found a complex phylogeographical history that supported continuous gene flow across southern Europe. Previously published carnivore studies, including those of brown bears (Valdiosera et al., 2007) and grey wolves (*Canis lupus*; Pilot et al., 2010), have not included material from the British Isles, and so it is still unclear whether some ecologically adaptable species could have survived as far north during the glacial cycles.

The red fox (*Vulpes vulpes* L.) is the most widely distributed terrestrial mammal, with a natural range extending across the entire Holarctic and an introduced range including Australia (Schipper et al., 2008). Red foxes are one of the most evolutionarily plastic species, being found in habitats ranging from tundra to deserts, although the breadth of the red fox niche could reflect differential local adaptation rather than phenotypic plasticity within populations (Sacks et al., 2010). Despite clear fossil evidence of red fox in continental Europe and England from MIS4 onwards (*c*. 71,000 years ago) to modern times (Sommer and Benecke, 2005a; Sommer and Nadachowski, 2006), only recently has fox phylogeography been examined. To date, studies in Japan (Inoue et al., 2007) and North America (Aubry et al., 2009) have demonstrated the importance of glacial cycles on the phylogeographic structure of the red fox. In Europe, studies have been restricted to the Mediterranean basin (Frati et al., 1998), or based on small sample sizes from archaeological remains found in northern and central Europe (Teacher et al., 2011). Although it seems clear from these studies that European red foxes appear to comprise a large population that has been relatively stable over the past 40,000 years (Teacher et al., 2011), considerably larger sample sizes are needed to characterise post-Pleistocene phylogenetic patterns.

In this study, our principal aim was to examine whether the red foxes in the British Isles were derived from a late Pleistocene colonisation from mainland Europe or whether they might reflect an ancient lineage that persisted in the region throughout the later ice ages. We also examined the phylogeographic and demographic patterns in the western Palaearctic region as a whole, to characterise more fully the effects of Quaternary climatic changes on extant red fox populations throughout Europe.

## 2 Materials and methods

### 2.1 DNA extraction and amplification

#### 2.1.1 Modern samples

**Fig. 1:**
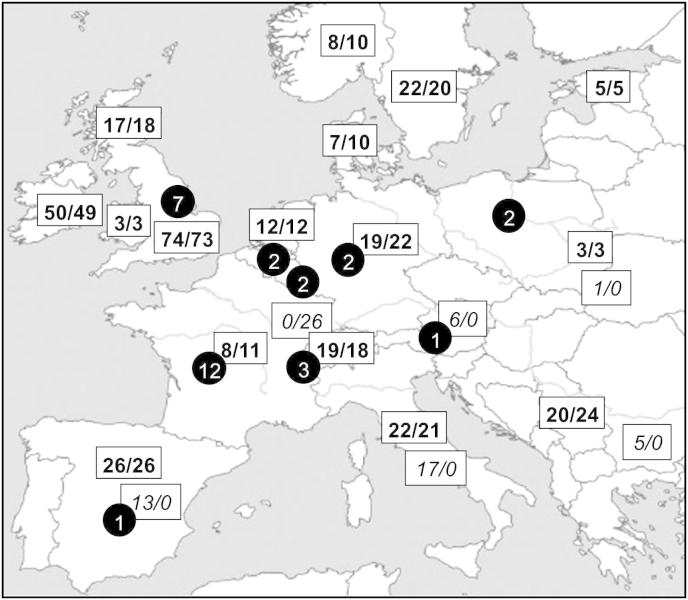
Map of sample locations. Numbers indicate modern cytochrome *b* data/modern control region data, with numbers in bold denoting sequence data generated as part of this study and numbers in italic showing published data used for analysis (for more details see Table S1). Black circles indicate ancient sequences, with sample sizes.

We collected 331 tissue samples from the following regions across Europe: Denmark, England, Estonia, France, Germany, Holland, Ireland, Italy, Norway, Poland, Serbia, Scotland, Sweden, Switzerland and Wales (Fig. 1, Table S1). Total genomic DNA was isolated using the DNeasy Blood and Tissue Kit (QIAGEN Ltd.), following the manufacturer's instructions. DNA from 242 modern samples was extracted, amplified and sequenced at Trinity College Dublin (TCD). Two regions of the mitochondrial genome were studied: a 464 bp fragment, including 422 bp of the 5′ end of the cytochrome *b* (*cytb*) gene, was amplified using the primers from Frati et al. (1998), and a 402 bp fragment, including 325 bp of the 5′ end of the control region (CR), was amplified using the primer pair JP-VVF (5′-CTC CCT AAG ACT CAA GGA AG-3′) and JP-VVR (5′-CCT GAG GTA AGA ACC AGA TGC-3′), designed as part of this study. The 50 μl reaction mixture contained 5 μl of template DNA, 2.5 Units Platinum^®^ *Taq* DNA Polymerase (Invitrogen), 2.5 mM MgCl_2_, 50 mM KCl, 20 mM Tris–HCl (pH 8.4), plus dNTPs at 0.2 mM, and 0.4 mM of each primer. The PCR program consisted of an initial denaturation step of 4 min at 94 °C, followed by 35 cycles of 94 °C for 30 s, 55 °C for 30 s, 72 °C for 30 s, and a final elongation step of 4 min at 72 °C. PCR amplicons were purified using the QIAquick PCR purification kit (QIAGEN Ltd.) according to the manufacturer's instructions, and sequencing was performed by Macrogen Inc. (*www.macrogen.com*).

A subset of 89 samples was analysed at the University of California, Davis. The 5′ portion of *cytb* was amplified using the primers RF14724 and RF15149 and conditions published by Perrine et al. (2007), and a 409 bp fragment of the CR using primers VVDL1 and VVDL6 and conditions from Aubry et al. (2009). PCR products were purified using ExoSAP-IT (USB/Affymetrix Inc.) and sequenced in both forward and reverse directions on an ABI 3730 capillary sequencer (Applied Biosystems). Sequencher version 4.8 (Gene Codes, Inc.) was used to visualise chromatograms and to edit and align sequences.

#### 2.1.2 Archaeological samples

Remains from two ancient red foxes were obtained from Carsington Pasture Cave in Derbyshire, England (a left ulna: CPC-98-2251, and a right radius: CPC-98-2252). DNA extractions from ancient material were performed as described in Edwards et al. (2010) and amplification of mitochondrial DNA was performed as described above for the TCD analysis. Appropriate measures were followed to control contamination (Cooper and Poinar, 2000) and multiple controls were used (Spencer and Howe, 2004). No contaminant bands were observed during any part of the analyses and, to identify possible post-mortem damage in the ancient DNA, sequencing was undertaken in both forward and reverse directions from several PCR products derived from two independent extractions. In addition, ancient DNA data from Austria, Belgium, England, France, Germany, Luxembourg, Poland, Spain and Switzerland were included from the study by Teacher et al. (2011): 30 *cytb* and 27 CR sequences (Fig. 1) were used in the network analysis, while 20 stratigraphically dated samples were included in the Bayesian analysis.

### 2.2 Phylogenetic analysis

#### 2.2.1 Statistical analysis

Thirty-six mitochondrial *cytb* sequences from Frati et al. (1998) were included (eight populations from Austria, Bulgaria, Italy, Sardinia and Spain), as well as four Galician (GenBank accession numbers AY586403–AY586406), and one Ukrainian (Fernandes et al., 2008) sequence. For the analyses of the mitochondrial CR, 26 sequences from France/Switzerland (Valiere et al., 2003) were included. Except for the ancient DNA samples from Teacher et al. (2011), which were shorter fragments, this gave a total long-fragment dataset of 356 *cytb* sequences and 351 CR sequences. All mitochondrial DNA sequences were aligned by ClustalX (version 1.8; Thompson et al., 1997) and were truncated in order to include the maximum number of published and new sequences, leaving 321 bp of the 5′ end of *cytb* and 251 bp of the 5′ end of the CR. Median networks were constructed using the median-joining algorithm of Bandelt et al. (1999) in Network (version 4.6; *www.fluxus-engineering.com*).

Phylogenetic analyses of *cytb* and CR sequences were conducted separately. The ARLEQUIN software (version 3.5; Excoffier et al., 2005) was used to estimate haplotype and nucleotide diversity, and to generate a matrix of pairwise *F*_ST_ based on haplotype frequencies (Weir and Cockerham, 1984) and a matrix of pairwise *Φ*_ST_ to incorporate pairwise differences between haplotypes (Nei and Li, 1979). Pairwise *F*_ST_ values can be used as an estimate of genetic distances between populations over shallow time depths (Slatkin, 1995). Multidimensional scaling (MDS; Kruskal, 1964) was performed based on the pairwise *Φ*_ST_ matrices obtained using the SPSS^©^ program (Statistics, version 19). Statistical significance (*α* = 0.05) of *Φ*_ST_ was based on 110 permutations, and then corrected for multiple tests using the sequential Bonferroni method (Rice, 1989). The program SAMOVA (version 1.0; Dupanloup et al., 2002) was used to assess population structure in the two datasets. The analysis was run using 100 simulated annealing processes for *K* values from 2 to 14.

#### 2.2.2 Temporal network analysis

To allow comparison of the ancient DNA data taken from Teacher et al. (2011) to our modern results, the *cytb* sequence was truncated to 201 bp and the CR was truncated to 193 bp. These alignment lengths differ from the Network analysis above as the latter did not include any ancient data. Two alignments were constructed: 386 individuals for *cytb*, comprising 355 modern and 31 ancient (30 from Teacher et al., 2011; plus our CPC individual); and 378 individuals for the CR, comprising 351 modern and 27 ancient (all from Teacher et al., 2011). For each of the two alignments, sequences were divided into three time categories: modern, Holocene (1500–12,000 years ago), and Pleistocene (>12,000 years ago). To assess the continuity of haplotypes across time, a temporal network was constructed from each sequence alignment using TempNet (Prost and Anderson, 2011).

#### 2.2.3 Bayesian analysis using modern and ancient data

To investigate phylogeographic patterns and to estimate the timescale of events, Bayesian analysis was performed on a concatenated alignment with BEAST (version 1.6.1; Drummond and Rambaut, 2007). This combined *cytb*/CR dataset contained 394 bp in total (201 bp of *cytb* and 193 bp of the CR), with 301 modern and 20 ancient red foxes. First, selection of an appropriate substitution model and partitioning scheme was performed using the Bayesian information criterion (BIC) in PartitionFinder (Lanfear et al., 2012). The best scheme was one in which separate substitution models were assigned to *cytb* (K80 + G) and to the CR (TIMef + G). This scheme had a better BIC score than schemes in which the two regions were grouped together, or in which the different codon positions in *cytb* were treated separately. Rate heterogeneity among sites was modelled using a discrete gamma distribution with six rate categories (Yang, 1994). The analysis was repeated without a model of rate variation among sites.

The data were analysed using a discrete phylogeographic model, allowing rates of pairwise spatial diffusion to be estimated (Lemey et al., 2009). Sequences were grouped into seven discrete geographic categories: Ireland, Britain, Iberia, Central Europe, Scandinavia, Italy, and the Balkans. Forward and backward diffusion rates between each pair of locations were allowed to differ, while non-zero diffusion rates were identified using Bayes factors (with a cut-off value of 10.0). All diffusion rates were assumed to be equally likely, such that geographic distance did not inform their prior probabilities.

To place a timescale on the estimates of population history, phylogeny, and phylogeographic history, the molecular clock was calibrated using the stratigraphic dates from Teacher et al. (2011) and the radiocarbon date from the Carsington Pasture Cave specimen. To test whether the structure and spread of these sampling times were sufficient for calibration, five replicates were analysed in which the dates were randomised among the sequences using SiteSampler (Ho and Lanfear, 2010). The 95% credibility intervals of the estimated rates did not include the mean estimate from the original, non-randomised dataset (Fig. S1), indicating that the sampling times of the sequences were sufficient for calibration (Firth et al., 2010; Ho et al., 2011). Posterior distributions of parameters were estimated using MCMC sampling. Samples were drawn every 10,000 MCMC steps over a total of 100,000,000 steps, with the first 10% of samples discarded as burn-in. The results of two independent analyses were compared to check for convergence to the stationary distribution, and samples from the posterior were combined. Sufficient sampling was checked using Tracer (version 1.5; *http://tree.bio.ed.ac.uk/software/tracer*).

Large differences in geographic representation between ancient and modern datasets can lead to biased estimates of substitution rates (Navascués and Emerson, 2009). To investigate this potential problem in our data on our rate estimates, we analysed the geographic subset for which we had sufficient numbers of both ancient and modern samples, specifically Central Europe. This dataset included samples from Austria (1 ancient), Belgium (1 ancient), France (8 modern and 7 ancient), Germany (17 modern and 1 ancient), Holland (12 modern), Poland (3 modern and 2 ancient) and Switzerland (18 modern and 3 ancient).

#### 2.2.4 Modern demographic analyses

To test demographic hypotheses and quantify isolation between mainland Europe and Britain and Ireland, Markov chain Monte Carlo (MCMC) simulations were conducted using IMa (Hey and Nielsen, 2007). Both ‘isolation with migration’ and ‘isolation with no migration’ models were tested and maximum-likelihood estimates produced, with 90% highest posterior density intervals for splitting times, effective population sizes and, depending on the model, gene flow in each direction. To minimise substructure within the two populations, the mainland European dataset was restricted to sequences from Central Europe, resulting in a comparison between 139 British and Irish sequences and 55 Central Europe sequences. The HKY substitution model was used. These analyses involved mutation-scaled parameters and were, therefore, independent of the mitochondrial mutation rate, which was unknown. However, it was necessary to assume particular mutation rates to translate these scaled estimates into biologically meaningful quantities. This was done using both evolutionary rates consistent with those inferred for, and supported independently in, multiple canid species (for example, Vilà et al., 1997; Wayne et al., 1997; Savolainen et al., 2002; Aubry et al., 2009), and an internal-node based estimate in the present study derived from the Bayesian analysis with ancient and modern samples described above (see also Teacher et al., 2011). In particular, the evolutionary rate for the combined *cytb* and CR fragment used in this study was assumed to be 9.36% per million years (Myr), which was the weighted average of mutation rates for the *cytb* (2.8%; 321 bp) and CR (17.75%; 251 bp) portions (Vilà et al., 1997; Wayne et al., 1997; Savolainen et al., 2002; Aubry et al., 2009).

After several initial runs with default parameters, 60 geometrically heated chains (Hey and Nielsen, 2007) were used with parameter ‘-g1’ set to 0.9. Multiple runs of 1000 steps (following 104 or 105 iterations as burn-in as necessary) were conducted to assess mixing and to narrow the range of parameter space. A second independent (different random seed) run of 106 steps in MCMC mode was then conducted. Consistent marginal peak locations (parameter estimates) with unimodal likelihood curves approaching zero on both ends, effective sample sizes of >50, and trend plots free of systematic change indicated good mixing. To produce final parameter estimates based on joint distributions, the ‘LoadTree’ mode in IMa was employed using trees produced in the longer MCMC run (Hey and Nielsen, 2007).

Inferences regarding the occurrence of past events of population expansion or decline were based on neutrality test estimates, as calculated via Tajima's *D* (Tajima, 1996) and Fu's Fs (Fu, 1997) in ARLEQUIN (version 3.5; Excoffier et al., 2005). Values were calculated for all modern *cytb* and CR samples, and for each region separately.

## 3 Results

We generated 314 modern *cytb* sequences and 325 CR sequences for this study (Fig. 1). For the *cytb* analysis, a further 42 sequences from European foxes were taken from published (Frati et al., 1998) and archived (GenBank accession numbers AY586403–AY586406) sources. Of the two archaeological fox samples from Carsington Pasture Cave, CPC-98-2251 yielded a reproducible reliable sequence using the *cytb* primer set of Frati et al. (1998), but no result from the CR primer set. This sample was radiocarbon dated to 4712 ± 22 uncalibrated radiocarbon years (UBA-14767). This date equates to 5325–5578 years calibrated BP, so this English fox dates to the Neolithic, which arrived in Britain about 4000 cal. years BC (Brown, 2007). No DNA was amplifiable from CPC-98-2252. Twenty-six CR sequences from the region of France/Switzerland were also incorporated into the analysis (Valiere et al., 2003). Thus the long-fragment dataset included 356 individuals with *cytb* sequences, 351 individuals with CR sequences and 301 individuals with both fragments. The short-fragment dataset included the same concatenated sequences (trimmed) along with 30 additional published ancient sequences (Teacher et al., 2011).

### 3.1 Modern haplotypes across Europe

**Fig. 2:**
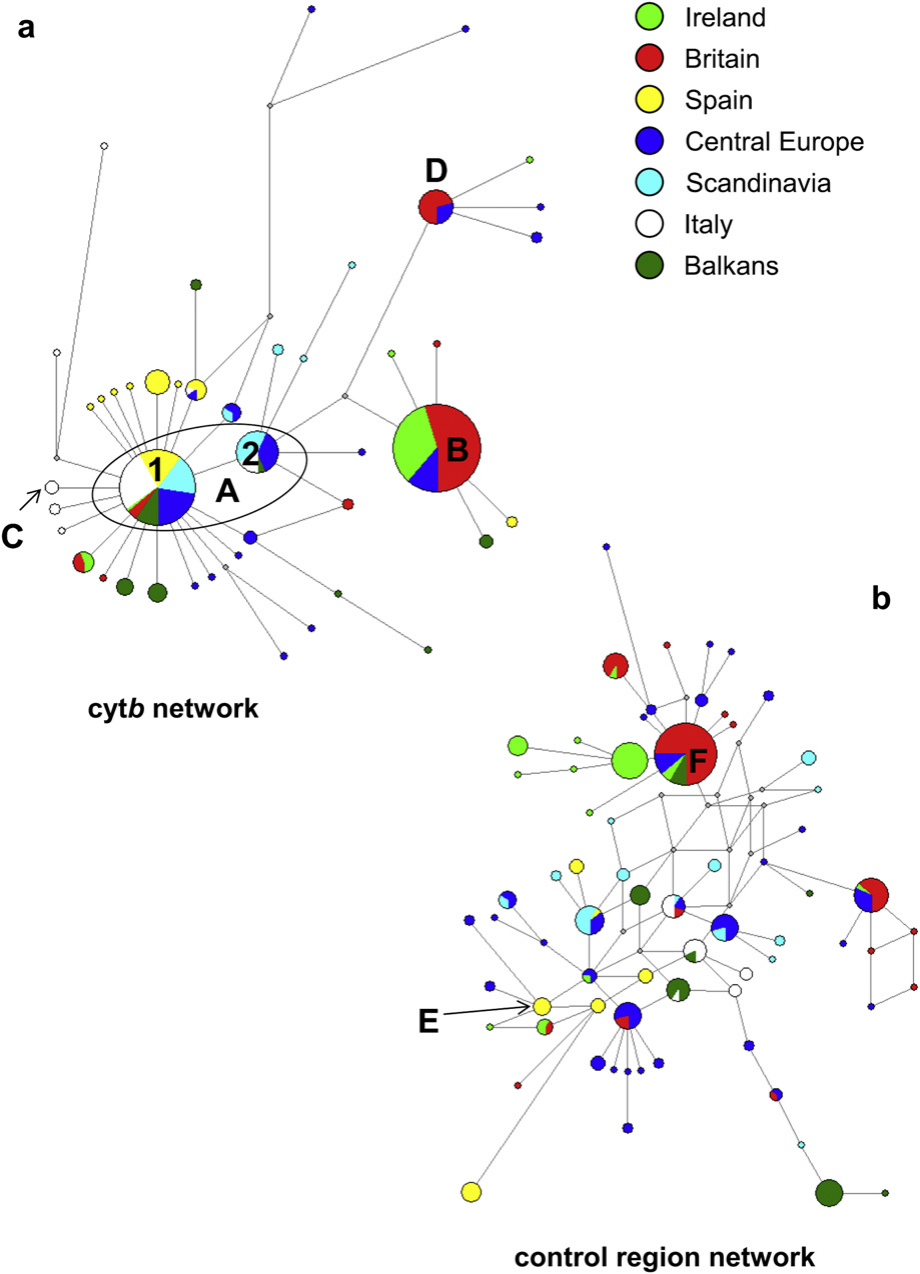
Median networks constructed from (a) 321 bp of *cytb* from 356 modern red fox sequences, and (b) 251 bp of CR from 351 modern red fox sequences, across Europe. Circles represent sequence haplotypes, the area being proportional to the frequency of the haplotypes. Points are theoretical intermediate nodes introduced by the median-joining algorithm, and branches between haplotypes represent single nucleotide mutations. Haplotypes labelled with a letter are discussed in the main text and equal those shown in Fig. 5 and Table 3.

Based on the long-fragment dataset, 44 *cytb* and 69 CR haplotypes were found in Europe, of which only seven and 15, respectively, were found in multiple locations (Fig. 1, Tables 1 and 2). Of the four most common *cytb* haplotypes, only haplotype A1 showed a wide geographic spread, being found in all regions (Fig. 2a). The remaining haplotypes were generally common in one or two regions: haplotypes B and D (Fig. 2a) were mainly found in the British Isles (Ireland and Britain, at 88.8% and 71.4% respectively), whereas haplotype A2 was predominantly found (76.7%) in Scandinavia and central Europe. In contrast to *cytb*, the CR haplotypes were more differentiated and no single haplotype was found in all regions. Only four were found in more than two regions, and most were restricted to one or two populations (Fig. 2b).

### 3.2 Population structuring and glacial refugia

**Fig. 3:**
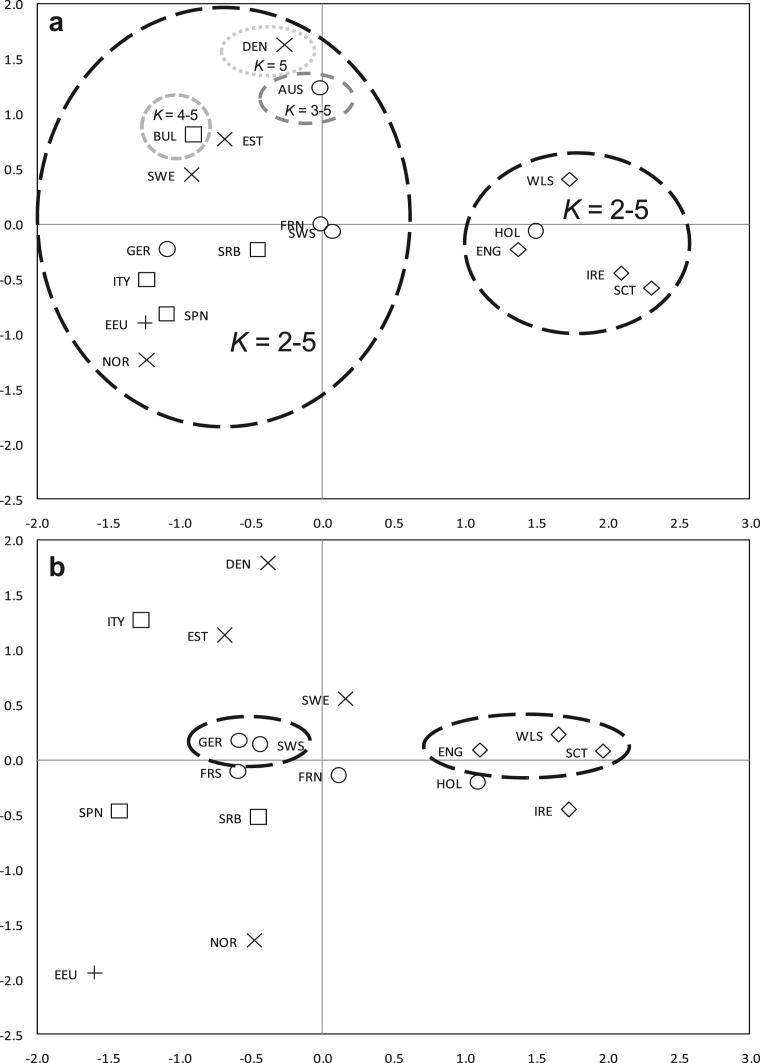
Two-dimensional MDS plots, drawn using data from: (a) 321 bp of *cytb*, and (b) 251 bp of CR, summarising genetic distances among the modern red fox populations. (a) The proportion of the data explaining the first two principal coordinates, the *R*^2^ value, is 87.0%. Hierarchical groupings as calculated by SAMOVA (*K* values 2–5) are shown, highlighting the independence of the Holland and British Isles cluster to increasing values of *K*. (b) The proportion of the data explaining the first two principal coordinates, the *R*^2^ value, is 82.6%. For this rapidly mutating marker, the best hierarchical grouping improves as *K* is increased, until *K* = 14, where only neighbouring Germany and Switzerland, and the British Isles cluster together (as shown).

All *Φ*_CT_ values produced by SAMOVA were statistically significant (data not shown). The highest *Φ*_CT_ value for the *cytb* dataset corresponded to *K* = 4 (Table S2), but two of these groups contained a single population, that of Austria and Bulgaria, both taken from the study by Frati et al. (1998). The highest configuration with no single population groupings was *K* = 2. The geographical organisation of these groups showed clustering of the British Isles (Britain and Ireland) with Holland, and all other populations forming the other group. The grouping of Holland and British Isles was consistent across all values of *K* (Fig. 3a). At *K* = 7, samples from Estonia, Denmark and Sweden clustered separately from mainland Europe and Norway; this clustering pattern was also supported by pairwise *F*_ST_ values (Table S3).

The highest *Φ*_CT_ value produced by SAMOVA for the CR dataset was *K* = 14 (Table S2), suggesting a high degree of differentiation based on this more rapidly mutating marker. The highest configuration with no single population groupings was, again, *K* = 2. However, the best hierarchical grouping improved as *K* increased, until *K* = 14, where only neighbouring Germany and Switzerland, and the British Isles (Britain and Ireland) clustered together (Fig. 3b). Pairwise *F*_ST_ values showed a similar pattern (Table S4).

### 3.3 Distribution of haplotypes through time

Three *cytb* haplotypes were found in the Pleistocene, Holocene and modern-day eras (Fig. 5a). These were split broadly across samples from Britain and mainland Europe (Table 3). Two of these haplotypes were also the most common ones found in modern samples (Fig. 2a); the geographic distribution of these Pleistocene and Holocene haplotypes is very similar to the modern day haplotype distribution. In particular, haplotype B is found most frequently in the British Isles, Holland and France, and is also found in these regions during the Pleistocene and Holocene, suggesting constant occupation during the LGM. In contrast, no CR haplotypes were found across all eras, although a small number were found in two different eras (Fig. 5b). This may partly reflect high genetic differentiation among locations, but also likely reflects sampling error given the high diversity of this marker in all locations.

### 3.4 Bayesian analysis of modern and ancient data

**Fig. 4:**
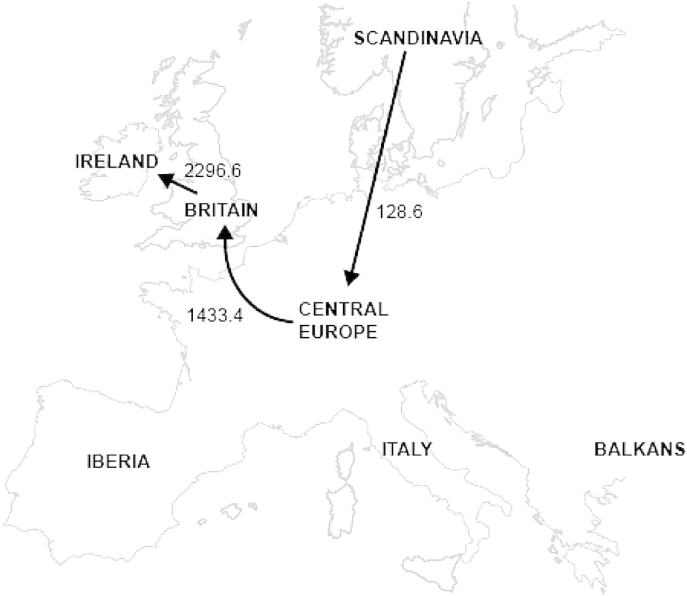
Map describing the seven locations (Balkans, Britain, Central Europe, Iberia, Ireland, Italy and Scandinavia) to which each of the 301 modern and 20 ancient red fox were assigned in the combined *cytb*/CR phylogeographic analysis. Non-reversible diffusion rates were estimated across the entire distribution of posterior trees and, therefore, reflect average rates of diffusion over time. Pairwise diffusions with Bayes factor >10 are shown; these are considered to be different from zero.

**Fig. 5:**
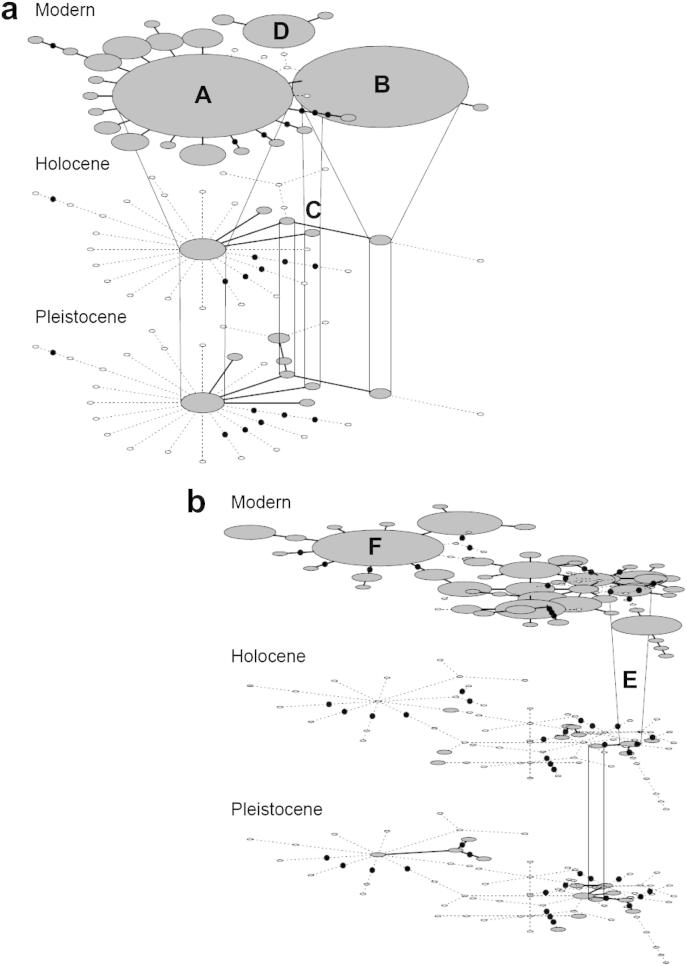
Temporal networks constructed from: (a) 201 bp of *cytb* from 355 modern and 31 ancient red foxes, and (b) 193 bp of CR from 351 modern and 27 ancient red foxes. Haplotypes labelled with a letter are discussed in the main text and equal those shown in Fig. 2 and Table 3.

Bayesian phylogenetic analysis using a discrete geospatial model showed that the most highly supported pairwise diffusions were from Britain to Ireland, Central Europe to Britain, and Scandinavia to Central Europe (Fig. 4). The posterior median coalescence time for all lineages was 50,000 years, with a 95% credibility interval of 32,700–77,800 years. The posterior median mutation rates estimated for our 394 bp *cytb*/CR fragment based on the Central European ancient and modern samples were 33.81% per Myr (95% CI: 13.75–59.38% per Myr) or 26.29% Myr (95% CI: 16.12–41.54% per Myr) depending, respectively, on whether the model included gamma-distributed rates among sites or assumed a constant rate among sites.

### 3.5 Demography

Tests of neutrality based on *cytb* revealed statistically significant evidence of population expansion only in Iberia and Italy, although both Fu's F and Tajima's *D* statistics were consistently negative, suggestive of a weak signature of expansion throughout Europe (Table 1). Tests of neutrality based on the more rapidly mutating CR revealed statistically significant evidence of population expansion only in Central Europe, according to a strongly negative Fu's F value. Otherwise, values varied from weakly and non-significantly negative to positive across regions, revealing no clear trend (Table 2).

Hypotheses of demographic isolation between mainland Europe and the British Isles were tested using IMa (Hey and Nielsen, 2007). Assuming a nucleotide mutation rate of 9.36% per Myr, both the ‘isolation with migration’ and ‘isolation only’ models indicated that the red fox populations in the British Isles became isolated from those of mainland Europe around the end of the last ice age (Table 4). Although the model incorporating migration suggested a somewhat earlier split than the model assuming no migration, both models have a lower 90% HPD limit around 6000 years. The isolation with migration model indicated some degree of gene flow since colonisation from mainland Europe to Britain and Ireland but not in the reverse direction, where the posterior density function displayed a mode approaching zero. The higher mutation rates estimated from the Bayesian analyses imply a much more recent splitting time, more in line with a mid-Holocene divergence (Table 4).

## 4 Discussion

### 4.1 British Isles fox populations

Most British mammals show genetic affinity to the Eurasian mainland populations; for example, badgers (*Meles meles*; Marmi et al., 2006), red deer (*Cervus elaphus*; Sommer et al., 2008), and least weasel (*Mustela nivalis*; Lebarbenchon et al., 2010), but British red foxes are clearly diverged from mainland populations. Fossil evidence suggests that foxes were present in Britain around 28,000 to 35,000 years ago (Sommer and Nadachowski, 2006), and ancient fox samples (Teacher et al., 2011) support the presence of the predominant British *cytb* and CR haplotypes in England and Central Europe (France, Switzerland and Luxembourg) during the Pleistocene, through into the Holocene. The strongly supported Bayesian diffusion rates suggest that British foxes originated from these Central European populations and, prior to the formation of the English Channel, were part of a contiguous population. Gene flow would have been restricted as the channel was forming but, as evidenced by the SAMOVA grouping between Britain and Holland in modern samples, probably continued between these areas across Doggerland prior to full submersion of this landbridge around 8,200BP. Using the more traditional assumptions about mtDNA mutation rates (that is, based on model-adjusted divergence in closely-related species calibrated to the fossil record), the IMa analysis indicates that British fox populations underwent isolation and subsequent divergence of haplotypes with the closure of the landbridge. This evidence lends support to a founder event and subsequent independent evolution in isolation in Britain.

There is contradictory evidence with respect to the relative origins of Irish and British foxes. As can be seen from the high number of *cytb* haplotypes shared between the two countries, these populations would seem to share a common origin, but less clear is which population was established first or from the other. On the one hand, the Bayesian geospatial analysis provides support for an origin of Irish foxes from British ones. On the other hand, the CR network indicates that Irish foxes have a high frequency (the majority) of unique haplotypes, most of which form a unique clade with haplotypes up to three mutations removed from the basal haplotype shared with Britain. British foxes have an extremely low frequency of unique haplotypes, comprising three singletons, each one mutation away from a haplotype shared with Ireland and mainland Europe.

Independent evidence is also inconclusive. Red foxes are assumed to be native to Ireland, but direct Pleistocene fossil evidence for their presence is lacking (Yalden, 1999). Instead, fossil records indicate that red foxes were first recorded between 5000 and 3000 BP (Sommer and Benecke, 2005a), after the closure of a putative landbridge to Ireland around 9500 years ago (Wingfield, 1995). However, overland colonisation of Ireland did occur in some species, including the brown bear (Sommer and Benecke, 2005b) and stoat (*Mustela erminea*; Martínková et al., 2007). Thus, despite the lack of fossil evidence for their ancient presence in Ireland, it is possible that foxes colonised Ireland independently during the Pleistocene, and then colonised Britain from there. If, instead, red foxes only arrived in Ireland (from Britain or elsewhere) in the mid-Holocene, the CR data paint a misleading picture. Clearly this question would benefit from the addition of other genetic markers because the mitochondrial markers used in the present study provide only a single genealogical reflection of the demographic history of these populations.

### 4.2 Phylogeographic structuring of red foxes

Our findings were consistent with those of previous studies (Frati et al., 1998; Teacher et al., 2011) in revealing a lack of deep phylogenetic structure, such as on the scale of that seen in North American red foxes, where major lineages diverged on the order of 400,000 years ago (Aubry et al., 2009). Nevertheless, our use of a relatively large dataset with a broad geographic coverage of Europe enabled us to elucidate population structure and historical demography in this region. Both *cytb* and CR data provided evidence of structuring of populations across Europe. Most notable was the division between most of Europe and Britain, Ireland and Holland, for example as evidenced in the MDS plots. Also evident from *Φ*_ST_, MDS, SAMOVA, and Bayesian diffusion analyses was a relatively high degree of isolation of Iberian foxes from those of other European regions and, more generally, less connectivity in the southern than northern regions. This finding was consistent with those of Frati et al. (1998), who found relatively high differentiation across Mediterranean populations. Our findings of population structure, along with palaeontological evidence (Sommer and Benecke, 2005a; Sommer and Nadachowski, 2006) and ancient DNA analyses showing isolation by distance (Teacher et al., 2011), indicate that foxes were present across Europe throughout the last glaciation. A corollary of this inference is that red foxes were not restricted to peninsular refugia during the last ice age. Our results are in concordance with other studies, such as for brown bears (Valdiosera et al., 2007), a species that at one time was (incorrectly) believed to show structuring into peninsular refugia (Taberlet et al., 1998). Further evidence can be taken from the spatio-temporal stability of haplotypes. For example, *cytb* haplotypes found in areas such as France and England during the Pleistocene were found there subsequently in the Holocene, as well as in modern samples (see also Teacher et al., 2011).

Mitochondrial DNA variation of the red fox in Europe seems to indicate an expansion in population size. The occurrence of a historic bottleneck and subsequent expansion is further supported by the low nucleotide diversity in the *cytb* region (0.007), and CR (0.019), values similar to those seen in North American red foxes (0.009 and 0.023 respectively; Aubry et al., 2009). Tests of neutrality showed evidence of population expansion in the majority of regions; our data are drawn from a wider geographic area and have a larger sample size than previous studies, providing greater power to detect population change.

In areas covered by glaciers, we would expect to see patterns of colonisation. In Scandinavia, there appears to have been bi-directional recolonisation. Scandinavian populations showed two distinct structures; Denmark and Sweden appeared to cluster together with Estonia (mainly haplotype A2), whilst Norway apparently clustered with mainland European populations (all haplotype A1). However, a larger sample size is needed, especially as Norwegian foxes are from one peninsula in Finnmark, whilst Swedish and Danish foxes were more widely sampled. In addition, significant Bayesian diffusion rates suggest movement from Scandinavia to Central Europe, but not *vice versa*. Although more genetic evidence is needed from haplotypes in Russia and more easterly populations, this does suggest that foxes colonised Scandinavia from two different directions. This easterly and westerly colonisation into Scandinavia is observed in a range of species, including brown bears (Taberlet et al., 1998; Korsten et al., 2009), field voles (*Microtus agrestis*; Jaarola et al., 1999) and common shrews (*Sorex araneus*; Lundqvist et al., 2011), leading to a distinctive suture zone of haplotypes, as would seem evident in our *cytb* data. CR data are less clear as it shows much greater population differentiation. However, pairwise *F*_ST_ values also exhibit some evidence of clustering in these regions.

## 5 Conclusions

Our research relates to two broader themes. Firstly, we challenge the standard hypotheses of glacial refugia where mammals were restricted to refugial areas in the ice age. Species that were ecologically adaptable, such as the red fox, could persist outside these areas and now exhibit high diversity and population structuring consistent with a constant occupation of Europe during the LGM and Quaternary glacial cycles. Secondly, our results show the importance of a wide sampling strategy, and highlight the need for inclusion of multiple data sources, including fossil evidence, to strengthen conclusions drawn from modern datasets.

## Figures and Tables

**Table 1:** Sample size, number of mtDNA *cytb* haplotypes including singleton haplotypes, and gene diversity for each population. Tajima's *D* and Fu's Fs tests of selective neutrality are shown for each region.

Population	*n*	Haplotypes	Unique haplotypes	Singleton haplotypes	Haplotype diversity	Nucleotide diversity	Tajima's *D* (*P* value)	Fu's *F* (*P* value)
**Ireland**	50	5	2	2	0.26 ± 0.08	0.0028 ± 0.002	−1.36 (0.07)	−0.42 (0.43)
**Britain**							−0.47 (0.37)	−0.04 (0.53)
Scotland	17	2	0	0	0.12 ± 0.10	0.0011 ± 0.001		
England	74	7	3	2	0.51 ± 0.06	0.0051 ± 0.003		
Wales	3	2	0	0	0.67 ± 0.31	0.0062 ± 0.006		
**Iberia**							−1.45 (0.06)	−4.31 (0.01)
Spain	39	9	7	4	0.74 ± 0.05	0.0034 ± 0.003		
**Central Europe**							−1.17 (0.11)	−5.91 (0.02)
Holland	12	2	0	0	0.41 ± 0.13	0.0038 ± 0.003		
France	8	4	0	0	0.75 ± 0.14	0.0080 ± 0.005		
Switzerland	19	8	5	5	0.82 ± 0.06	0.0088 ± 0.005		
Germany	19	5	3	2	0.71 ± 0.08	0.0030 ± 0.002		
Austria	6	5	3	2	0.93 ± 0.12	0.0166 ± 0.011		
Denmark	7	2	0	0	0.29 ± 0.20	0.0009 ± 0.001		
**Scandinavia**							−0.67 (0.30)	−1.76 (0.11)
Sweden	21	5	3	2	0.69 ± 0.07	0.0030 ± 0.002		
Norway	8	1	0	0	0.00 ± 0.00	0.0000 ± 0.000		
Estonia	5	2	0	0	0.60 ± 0.18	0.0037 ± 0.003		
**Italy**	39	8	5	3	0.58 ± 0.09	0.0028 ± 0.002	−1.88 (0.02)	−3.98 (0.00)
**Balkans**							−0.56 (0.35)	−1.19 (0.27)
Bulgaria	5	4	3	2	0.90 ± 0.16	0.0087 ± 0.006		
Serbia	20	5	3	0	0.80 ± 0.04	0.0062 ± 0.004		
East Europe	4	1	0	0	0.00 ± 0.00	0.0000 ± 0.000		
**All samples**	**356**	**44**	**37**	**24**	**0.80 ± 0.02**	**0.0072 ± 0.004**	−**1.08 (0.18)**	−**2.52 (0.20)**

**Table 2:** Sample size, number of mtDNA CR haplotypes including singleton haplotypes, and gene diversity for each population. Tajima's *D* and Fu's Fs tests of selective neutrality are shown for each region.

Population	*n*	Haplotypes	Unique haplotypes	Singleton haplotypes	Haplotype diversity	Nucleotide diversity	Tajima's *D* (*P* value)	Fu's *F* (*P* value)
**Ireland**	49	12	7	5	0.71 ± 0.06	0.0110 ± 0.007	−1.11 (0.14)	−2.08 (0.21)
**Britain**							−0.47 (0.48)	−1.22 (0.36)
Scotland	18	4	1	1	0.31 ± 0.14	0.0060 ± 0.004		
England	73	11	5	5	0.68 ± 0.05	0.0126 ± 0.007		
Wales	3	2	1	1	0.67 ± 0.31	0.0106 ± 0.010		
**Iberia**							2.15 (0.98)	1.60 (0.78)
Spain	26	6	5	0	0.83 ± 0.03	0.0124 ± 0.007		
**Central Europe**							−0.31 (0.45)	−14.23 (0.00)
Holland	12	6	4	2	0.85 ± 0.07	0.0130 ± 0.008		
France	11	6	1	0	0.85 ± 0.09	0.0181 ± 0.011		
Switzerland	18	8	2	2	0.82 ± 0.06	0.0125 ± 0.008		
France/Switzerland	26	16	10	6	0.95 ± 0.02	0.0153 ± 0.009		
Germany	22	10	4	3	0.92 ± 0.03	0.0130 ± 0.008		
Denmark	10	3	0	0	0.38 ± 0.18	0.0060 ± 0.004		
**Scandinavia**							−0.63 (0.30)	−2.69 (0.12)
Sweden	20	10	7	3	0.91 ± 0.04	0.0140 ± 0.008		
Norway	10	2	1	1	0.20 ± 0.15	0.0040 ± 0.003		
Estonia	5	2	1	0	0.60 ± 0.18	0.0120 ± 0.009		
**Italy**	21	5	2	0	0.62 ± 0.09	0.0045 ± 0.003	0.94 (0.83)	0.34 (0.60)
**Balkans**								
Serbia	24	5	3	2	0.70 ± 0.06	0.0149 ± 0.009	1.23 (0.91)	3.34 (0.94)
East Europe	3	1	0	0	0.00 ± 0.00	0.0000 ± 0.000		
**All samples**	**351**	**69**	54	31	**0.93 ± 0.01**	**0.0186 ± 0.010**	**0.29 (0.58)**	−**2.14 (0.43)**

**Table 3:** Table detailing haplotype-sharing across modern, Holocene and Pleistocene time-slices. Haplotype labels correspond to those labelled in Fig. 2.

Haplotype label	Modern	Holocene	Pleistocene
A	Italy (31), Spain (21), Sweden (21), Germany (15), Serbia (11), Norway (8), Denmark (7), Switzerland (7), England (4), Estonia (3), Holland (3), Poland (3), Bulgaria (1), France (1), Ireland (1), Scotland (1)	France (7), England (1), Spain (1)	England (2), France (2), Austria (1), Belgium (1), Germany (1), Poland (1)
B	England (50), Ireland (44), Scotland (16), Holland (9), Serbia (3), Switzerland (3), France (2), Spain (2), Wales (2)	England (1), Switzerland (1)	England (1), France (1)
C	Italy (2)	Switzerland (1)	France (1)
D	England (14), Switzerland (4), Austria (1), France (1), Ireland (1), Wales (1)		Luxembourg (2)
E	France (2), Spain (6)	France (2)	
F	England (37), Scotland (15), Holland (8), Ireland (6), France (4), Serbia (4), Wales (2), Switzerland (1)		France (1)

**Table 4:** IMa joint estimates of demographic parameters: *N*_e_ (effective population size = *θ*/4*μ*; where *θ* is genetic diversity and *μ* is mutation rate), *N*_e_*m* (effective migration rate = *θm*/4; where *m* is the migration rate per generation), and population splitting times (*t*/*μ*) under two models based on concatenated *cytb* and CR sequences. The 90% highest posterior density (HPD) intervals corresponding to parameter estimates are indicated in parentheses. Unless otherwise indicated, estimates are based on the assumed site-specific mutation rate, 9.36% per Myr for the combined *cytb*/CR haplotype.

Demographic model:	Isolation with migration (90% HPD)	Isolation only (90% HPD)
*N*_e_ Britain/Ireland	150,000 (62,000–233,000)	215,000 (139,000–325,000)
*N*_e_ Central Europe	481,000 (243,000–1,035,000)	416,000 (225,000–844,000)
*N*_e_ Ancestral	112,000 (40,000–374,000)	262,000 (157,000–422,000)
*N*_e_*m* into Britain/Ireland	3270 (110–20,140)	–
*N*_e_*m* into Central Europe	–a	–
Splitting time	19,000 years (5900–51,500)	10,000 years (5700–14,500)
Alternative splitting time 1^b^	6765 years (2100–18,335)	3560 years (2030–5160)
Alternative splitting time 2^c^	5260 years (1630–14,260)	2770 years (1580–4015)

a The posterior density function smoothed peak approached zero for gene flow into Central Europe.

b These estimates assume a mutation rate of 26.29% per Myr. This value is based on the Bayesian median posterior estimate assuming homogeneous rates among sites, and does not take into account the 95% CI of the estimate.

c These estimates assume a mutation rate of 33.81% per Myr. This value is based on the Bayesian median posterior estimate using a gamma-distributed rate heterogeneity across sites, and does not take into account the 95% CI of the estimate.
