# Supplementary material for: Temporal genetic variation of the red fox, Vulpes vulpes, across western Europe and the British Isles
Source: Quat Sci Rev. 2012 Dec 4;57:95–104. doi: 10.1016/j.quascirev.2012.10.010 (PMC3778924; doi:10.1016/j.quascirev.2012.10.010)
Supplement: Supplementary file 1 [file mmc1.doc]

**Table S1.** Detailed location of the samples collected from across Europe, and other published data.

**Table S2.** Best groupings (indicated by letters) based on *cytb* and CR data sets for values of K from 2 to 14 groupings, using SAMOVA (Dupanloup et al., 2002).

**Table S3.** Pairwise *F*_ST_ and *Φ*_ST_ estimates based on *cytb* sequence data from the red fox populations sampled. Below diagonal, measures are based on haplotype frequencies (*F*_ST_); above diagonal, estimates incorporate pairwise differences in sequence divergence (*Φ*_ST_). Numbers underlined indicate statistical significance (α = 0.05) based on sequential Bonferroni correction for multiple tests (Rice, 1989).

**Table S4.** Pairwise *F*_ST_ and *Φ*_ST_ estimates based on CR sequence data from the red fox populations sampled. Below diagonal, measures are based on haplotype frequencies (*F*_ST_); above diagonal, estimates incorporate pairwise differences in sequence divergence (*Φ*_ST_). Numbers underlined indicate statistical significance (α = 0.05) based on sequential Bonferroni correction for multiple tests (Rice, 1989).

**Figure S1.** Bayesian rate estimate comparing the actual data (filled black circle) to rate estimates from five replicates in which the sample ages have been randomly shuffled. The rate estimates from the date-randomised replicates do not overlap with the original rate estimate, suggesting that there is sufficient information in the sample ages.
